# Wogonin Induces Apoptosis and Reverses Sunitinib Resistance of Renal Cell Carcinoma Cells *via* Inhibiting CDK4-RB Pathway

**DOI:** 10.3389/fphar.2020.01152

**Published:** 2020-07-24

**Authors:** Yong Wang, Shouzhen Chen, Shuna Sun, Guangyi Liu, Lipeng Chen, Yangyang Xia, Jianfeng Cui, Wenfu Wang, Xuewen Jiang, Lei Zhang, Yaofeng Zhu, Yongxin Zou, Benkang Shi

**Affiliations:** ^1^ Department of Urology, Qilu Hospital, Cheeloo College of Medicine, Shandong University, Jinan, China; ^2^ The Key Laboratory of Experimental Teratology of Ministry of Education, Department of Medical Genetics, School of Basic Medical Sciences, Cheeloo College of Medicine, Shandong University, Jinan, China; ^3^ Key Laboratory of Urinary Precision Diagnosis and Treatment in Universities of Shandong, Jinan, China; ^4^ Department of Dermatology, The Affiliated Hospital of Shandong University of Traditional Chinese Medicine, Shandong Provincial Hospital of Traditional Chinese Medicine, Jinan, China; ^5^ Department of Nephrology, Qilu Hospital, Shandong University, Jinan, China; ^6^ Department of Immunology and Key Laboratory of Infection and Immunity of Shandong Province, School of Basic Medical Sciences, Cheeloo College of Medicine, Shandong University, Jinan, China

**Keywords:** wogonin, RCC, apoptosis, CDK4-RB, sunitinib

## Abstract

Wogonin, an active component derived from Scutellaria baicalensis, has shown anti-tumor activities in several malignancies. However, the roles of wogonin in RCC cells remain elusive. Here, we explored the effects of wogonin on RCC cells and the underlying mechanisms. We found that wogonin showed significant cytotoxic effects against RCC cell lines 786-O and OS-RC-2, with much lower cytotoxic effects on human normal embryonic kidney cell line HEK-293 cells. Wogonin treatment dramatically inhibited the proliferation, migration, and invasion of RCC cells. We further showed that by inhibiting CDK4-RB pathway, wogonin transcriptionally down-regulated CDC6, disturbed DNA replication, induced DNA damage and apoptosis in RCC cells. Moreover, we found that the levels of p-RB, CDK4, and Cyclin D1 were up-regulated in sunitinib resistant 786-O, OS-RC-2, and TK-10 cells, and inhibition of CDK4 by palbociclib or wogonin effectively reversed the sunitinib resistance, indicating that the hyperactivation of CDK4-RB pathway may at least partially contribute to the resistance of RCC to sunitinib. Together, our findings demonstrate that wogonin could induce apoptosis and reverse sunitinib resistance of RCC cells *via* inhibiting CDK4-RB pathway, thus suggesting a potential therapeutic implication in the future management of RCC patients.

## Introduction

Renal cell carcinomas (RCCs), arising from the renal epithelium, account for approximately 85% of kidney cancers. Immunotherapy and targeted therapy are the recommended routine treatment strategies for patients with metastatic RCC (mRCC), but the therapeutic effects are disappointing (Ljungberg et al., 2015). Recently, several anti-angiogenic drugs especially tyrosine kinase inhibitors (TKIs), such as sunitinib and sorafenib, have been approved for its treatment. However, the efficacy of these drugs is generally limited by various resistance mechanisms (Buczek et al., 2014).

Wogonin, a natural flavonoid derived from Scutellaria baicalensis Georgi, has been reported to possess anti-tumour activities against various types of cancer (Baumann et al., 2008; Zhao et al., 2010; Yao et al., 2014) *via* different ways, such as increasing intracellular reactive oxygen species (ROS) (Qian et al., 2014), inducing apoptosis (Hu et al., 2015), arresting cell cycle (Lu et al., 2015) and reversing drug resistance (Kim et al., 2016). These multiple anti-tumor effects of wogonin could be related to the regulation of numerous cell signaling pathways, including serine-threonine kinase Akt and AMP-activated protein kinase (AMPK) pathways (Lee et al., 2012; Liu et al., 2016), p53-dependent/independent apoptosis and inhibition of telomerase activity (Huang et al., 2010b). However, whether wogonin possesses anti-tumour activities in RCC remains unknown.

In the present study, we assessed the anti-cancer effects of wogonin on RCC cells. We found that wogonin significantly inhibited proliferation, migration, and invasion of RCC cells. We further showed that by inhibiting CDK4-RB pathway, wogonin could down-regulate CDC6 and induce DNA replication defects, DNA damage, and apoptosis of RCC cells. Importantly, our data indicated that hyperactivation of CDK4-RB contributed to the resistance of RCC cells to sunitinib, and treatment with wogonin or palbociclib effectively reversed sunitinib resistance. Thus our findings suggest that wogonin is a promising agent in the future management of RCC patients.

## Materials and Methods

### Cell Culture and Manipulation

The human renal cancer cell line 786-O and human normal embryonic kidney cell line HEK-29 were obtained from the American Type Culture Collection (ATCC). OS-RC-2 cell line and TK-10 cell line were purchased from the Type Culture Collection of the Chinese Academy of Sciences, Shanghai, China. The cells were maintained in RPMI 1640 medium (Gibco, Grand Island, NY) supplemented with 10% fetal bovine serum (Hyclone, USA) plus 100 U/ml penicillin and 100 μg/ml streptomycin at 37°C in a humidified atmosphere with 5% CO_2_.

siRNA and plasmids transfections were performed using Lipofectamine 2000 (Life Technologies, CA, USA) according to the manufacturer’s instructions. siRNA targeting CDC6 and RB (siCDC6 and siRB) and negative control siRNA were purchased from GenePharman (Shanghai, China). Plasmid expressing HA-tagged full-length human CDC6 was a gift from L. Drury (Clare Hall Laboratories, Cancer Research UK, London, England) (Mailand and Diffley, 2005).

The sequence of siRNAs were listed in **Supplementary Table 1**.

Wogonin was purchased from Sigma-Aldrich (CAS: 632-85-9, purity ≥ 98%).

### MTT and Colony Formation Assays

MTT assays and colony formation assays were performed as described previously (Zou et al., 2013; Mi et al., 2017).

### Wounding Healing and Transwell Assay

Wounding healing assays and transwell assays were performed as previously described (Mi et al., 2017).

### Tumor Xenografts

Ten male BALB/c nude mice (4–6 weeks of age) were randomly divided into two groups: the control group and the wogonin group. After a week of adjustment, about 1×10^6^ 786-O/WT cells or 786-O/SR cells suspended in 100 μl PBS were injected subcutaneously into each mouse. Then the mice in wogonin group were given wogonin (40 mg/kg) intragastrically everyday for 2 weeks. Tumor volumes were measured every two days. The mice were then sacrificed, and the tumor weight and tumor size were measured. Animal handling and experimental procedures were approved by the Ethics Committee of Qilu Hospital of Shandong University.

### Cell Cycle Analysis

Cell cycle distribution was analyzed by flow cytometry as previously described (Zou et al., 2013).

### Immunofluorescence Staining

Immunofluorescence was performed as described previously (Zou et al., 2009). The antibodies are listed in **Supplementary Table 2**.

### Western Blot

Preparation of total and chromatin-bound protein extracts and Western blot analysis were performed as previously described (Zou et al., 2009). The primary antibodies are listed in **Supplementary Table 2**.

### Quantitative Reverse Transcriptase-Polymerase Chain Reaction (qRT-PCR)

Extraction of total RNA and qRT-PCR assays was performed as previously described (Zou et al., 2013). The primer sequences are listed in **Supplementary Table 3**.

### TUNEL and β-Galactosidase (SA-β-Gal) Activity Assay

Apoptotic cells were detected using one-step TUNEL Apoptosis Assay Kit (Beyotime, Beijing, China) according to the manufacturer’s instructions. After labeling, the slides were counterstained with DAPI and visualized under a fluorescence microscopy.

**C**ell senescence was detected using the Senescence β-Galactosidase Staining Kit (Beyotime, Beijing, China) according to the manufacturer’s instructions. Cells stained blue were regarded as positive.

### EdU Incorporation Assay

EdU (5-ethynyl-2′-deoxyuridine) incorporation assays were performed using the Cell-Light EdU DNA Cell Proliferation Kit (Ribobio, Guangzhou, China) according to the manufacturer’s instructions (Mi et al., 2017).

### Alkaline Comet Assay

Alkaline comet assays were carried out using Reagent Kit for Single Cell Gel Electrophoresis Assay (Trevigen, USA) according to the manufacturer’s protocol. Cells suspended in PBS at 1×10^5^ cells/ml were combined with molten LMAgarose (at 37°C) at a ratio of 1:10 (v/v). 50 μl mixture was then immediately pipetted onto Comet Slide™. After electrophoresis, the slides were stained with DAPI and viewed under a fluorescence microscope.

### Establishment of Sunitinib-Resistant Cell Lines

786-O, OS-RC-2, and TK-10 cells were continuously exposed to increasing dose of sunitinib up to a final concentration of 10 μM. Following continuous culture supplemented with 10 µM sunitinib for more than 10 passages, the cells were used as sunitinib-resistant RCC cell lines (786-O/SR, OS-RC-2/SR, and TK-10/SR) for subsequent experiments.

### TCGA Data Analysis of CDC6 Expression in RCC

The prognostic value of CDC6 expression in RCC using TCGA data of 522 renal clear cell carcinoma patients and 284 renal papillary cell carcinoma patient was analyzed by Oncolnc (http://www.oncolnc.org/). Kaplan–Meier survival curves were created in Oncolnc homepage.

### Statistical Analysis

Data were obtained from three independent experiments. All results are presented as the mean ± standard deviation (S.D.). All statistical analyses were performed using SPSS statistical software program (Version 13.0; SPSS Inc.). Differences between groups were analyzed by the Student t test or one way ANOVA. P < 0.05 was regarded as statistically significant.

## Results

### Wogonin Inhibits Proliferation, Migration, and Invasion of RCC Cells

**Figure 1:**
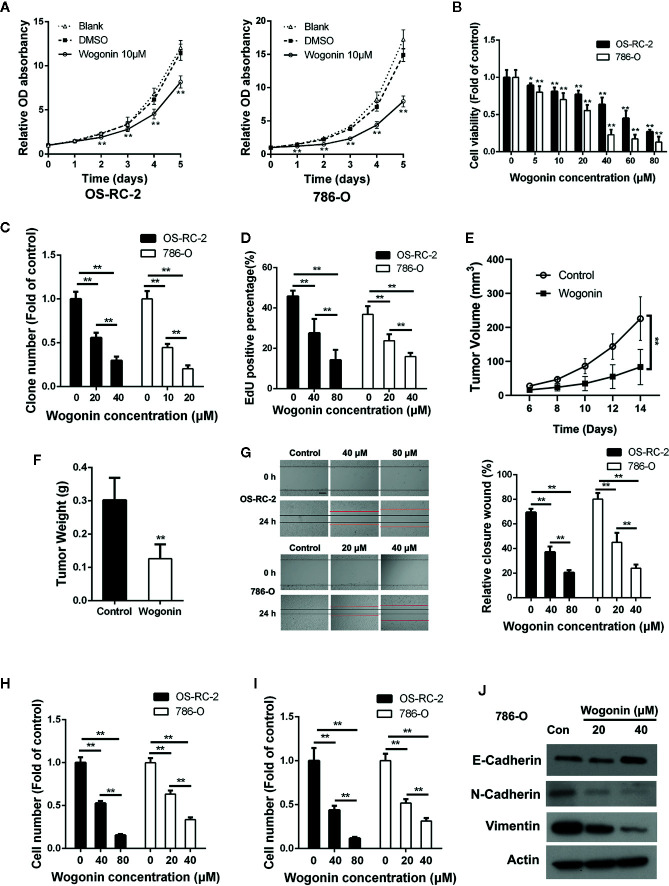
Wogonin inhibits proliferation migration and invasion of RCC cells. **(A)** MTT assays of OS-RC-2 and 786-O cells treated with or without 10 μM wogonin. **(B)** OC-RC-2 and 786-O cells were treated with wogonin at different concentrations for 48 h and the cell viability was determined by MTT assays. **(C)** Colony formation efficiency of OS-RC-2 and 786-O cells treated with or without wogonin. **(D)** EdU incorporation assays of OS-RC-2 and 786-O cells treated with or without wogonin for 24 h. **(E**, **F)** Tumor formation by 786-O cells in nude mice. 1×10^6^ 786-O cells were injected subcutaneously into nude mice. Mice were either treated with wogonin (40 mg/kg) or treated with saline (as control) everyday for 2 weeks. And tumor volumes were measure every 2 days **(E)**. Then the mice were sacrificed and the tumor weight **(F)** were measured. **(G)** Cells scraped with a pipette tip were treated with different concentrations of wogonin for indicated time and then the distances that the cells migrated were measured (scale bar, 200 μm). **(H, I)** Cells were treated with different concentrations of wogonin for 24 h, and transwell assays were performed to evaluate the ability of cell migration **(H)** and invasion **(I)** (scale bar, 50 μm). **(J)** 786-O cells were treated with indicated concentrations of wogonin for 24 h and the expression of E-Cadherin, N-Cadherin, and Vimentin were measured by Western Blot. *p < 0.05; **p < 0.01.

We first evaluated the anti-proliferative effects of wogonin on RCC cells. MTT assays showed that wogonin significantly reduced the growth of human RCC OS-RC-2 and 786-O cells (**Figure 1A**), and the proliferative inhibitory effects of wogonin were in a concentration-dependent manner (**Figure 1B**). Consistently, colony-formation assays showed that the wogonin treated RCC cells displayed much fewer and smaller colonies (**Figure 1C** and **Supplementary Figure 1A**). EdU incorporation assays were then conducted to investigate the effects of wogonin on DNA replication of RCC cells. As shown in **Figure 1D** and **Supplementary Figure 1B**, treatment of wogonin significantly decreased the percentage of EdU positive cells. However, MTT assays showed that inhibitive rate of wogonin at 80 μM on human normal embryonic kidney cell line HEK-293 cells was only about 20% compared with an inhibitive rate of more than 70% on RCC cells (**Supplementary Figure 1C**). Colony formation assay also showed that the effects of wogonin on inhibition of growth in HEK293 cells were much less evident (**Supplementary Figure 1D**), suggesting that HEK-293 cells were less sensitive to wogonin treatment than RCC cells.

Next we tested the effects of wogonin on tumor growth *in vivo* with a xenograft model. The results demonstrated that application of wogonin significantly suppressed tumor growth (**Figure 1E** and **Supplementary Figure 1E**). At the endpoint, the average tumor weight were significantly reduced in the group receiving wogonin (**Figure 1F**). Together, these results indicate that wogonin significantly inhibits the growth of RCC both *in vitro* and *in vivo*.

Previous studies reported that wogonin could inhibit migration and invasion of various cancer cells (He et al., 2013; Zhao et al., 2015). Wounding healing assays showed that treatment with wogonin significantly suppressed wound closure in a concentration-dependent manner in OS-RC-2 and 786-O cells (**Figure 1G**). In addition, the number of wogonin treated cells that migrated across transwell membrane was considerably lower than that of the control cells (**Figure 1H** and **Supplementary Figure 1F**). Furthermore, matrigel invasion assays also showed a great reduction in the number of invasive cells in wogonin treated group (**Figure 1I** and **Supplementary Figure 1G**). Together, these results indicate that wogonin significantly attenuate the ability of migration and invasion of RCC cells. Given the critical role of Epithelial-Mesenchymal Transition (EMT) in cancer cell invasion, we examined protein levels of several EMT markers by Western blot. As shown in **Figure 1J**, wogonin significantly decreased the expression of N-Cadherin and Vimentin, and increased the expression of E-Cadherin. The expression of Snail showed no difference after wogonin treatment (data not shown).

### Wogonin Induces Apoptosis of RCC Cells

**Figure 2:**
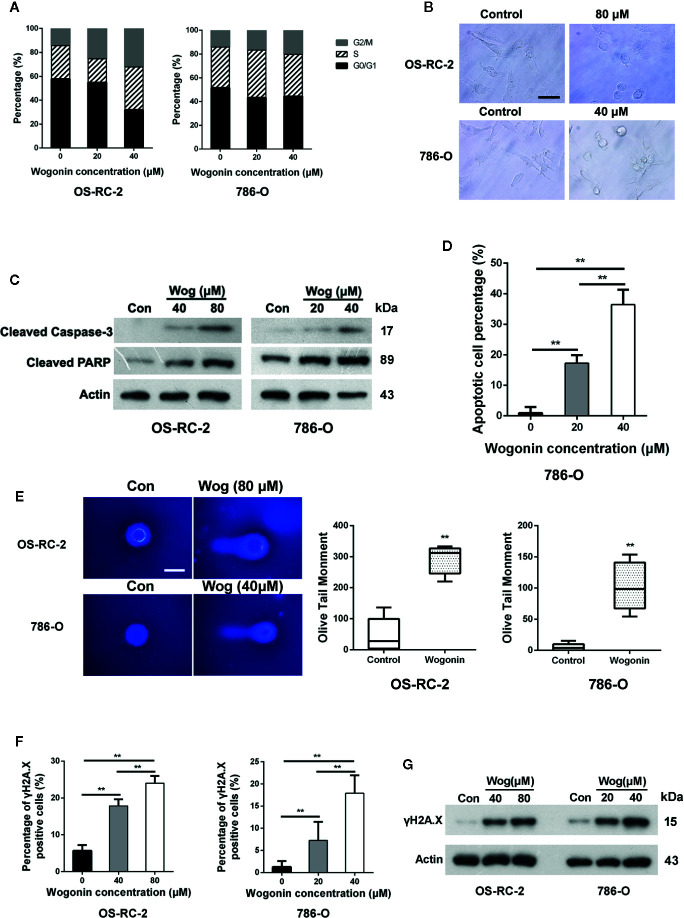
Wogonin induces apoptosis of RCC cells. **(A)** OS-RC-2 and 786-O cells were treated with or without wogonin for 48 h and cell cycle distribution were determined by flow cytometry. **(B)** The morphological change of OS-RC-2 and 786-O cells treated with or without wogonin for 48 h (scale bar, 50 μm). **(C)** OS-RC-2 and 786-O cells were treated with or without different concentrations of wogonin for 48 h and levels of cleaved caspase-3 and cleaved PARP were measured by Western blot. **(D)** TUNEL assays of 786-O cells treated with or without wogonin at different concentrations for 48 h. **(E)** Alkaline comet assays were performed on indicated cells treated with or without wogonin for 24 h (scale bar, 20 μm). **(F, G)** OS-RC-2 and 786-O cells treated with or without wogonin for 48 h and γH2A.X levels were determined by immunofluorescence **(F)** and Western Blot **(G)**. **p < 0.01.

To investigate the underlying mechanisms of cell growth inhibition caused by wogonin, OS-RC-2 and 786-O cells were treated with wogonin for 48 h and subjected to flow cytometric analysis. The results showed that the proportion of cells in G2/M phase increased accompanied by a decrease in G1 fraction in wogonin treated cells (**Figure 2A** and **Supplementary Figure 2A**).

We noticed that treatment of wogonin induced apparent morphological changes including membrane blebbing and reduced cell volume (**Figure 2B**), indicating that these cells were committed to cell death, such as apoptosis. Thus we measured the protein levels of cleaved caspase-3 and PARP by western blot. The results demonstrated that treatment of wogonin significantly increased the expression of these two proteins (**Figure 2C**). Consistently, the percentages of TUNEL positive cells were significantly increased by treatment with wogonin (**Figure 2D** and **Supplementary Figure 2B**). SA-β-gal activity assays were also performed, and no differences were observed in senescence between wogonin treated and control cells (**Supplementary Figure 2C**). Together, these data suggest that wogonin treatment induces G2/M phase arrest and apoptosis in RCC cells.

DNA damage has been shown to induce cell apoptosis (Norbury and Zhivotovsky, 2004). So alkaline comet assays were performed, and the results demonstrated that wogonin treatment significantly increased the mean olive tail moment value (**Figure 2E**). Expression of γH2A.X, a marker of DNA damage, was further measured using immunofluorescence staining and Western blot. The results showed that the levels of γH2A.X significantly increased in the cells treated with wogonin (**Figures 2F, G**). Together, these results indicate that wogonin induces DNA damage in RCC cells.

### Wogonin Down-Regulates Expression of CDC6 in RCC Cells

**Figure 3:**
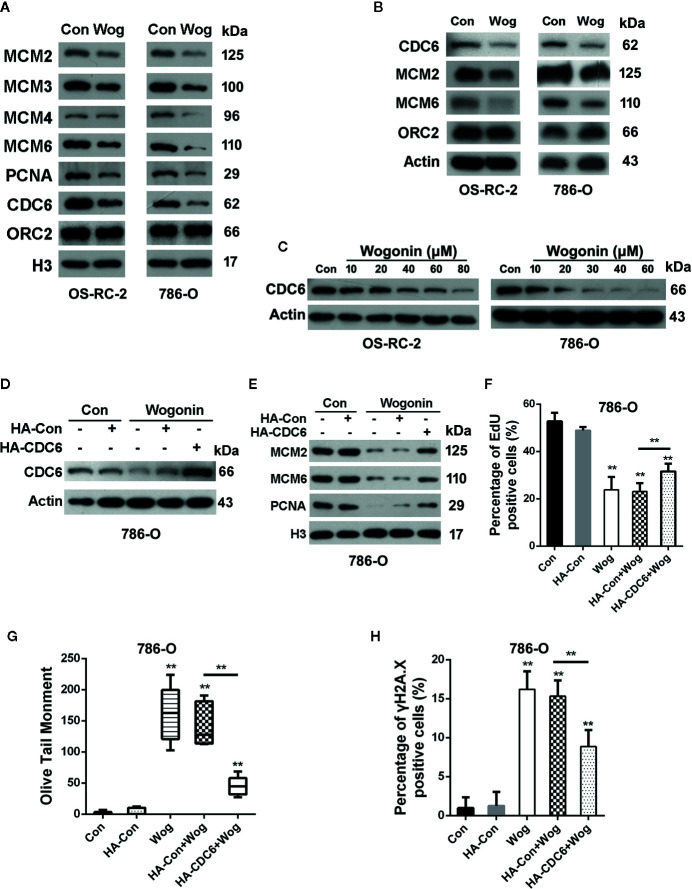
Wogonin down-regulates expression of CDC6 in RCC cells. **(A, B)** OS-RC-2 and 786-O cells were treated with or without wogonin at 40 μM and 20 μM respectively for 24 h and levels of indicated chromatin bound proteins **(A)** and total proteins **(B)** were measured by Western Blot. **(C)** Cells were treated with different concentrations of wogonin for 24 h and expression of total CDC6 was measured by Western blot. **(D**–**H)** 786-O cells were transfected with HA-tagged CDC6 expression plasmid or control plasmid and then treated with wogonin at 20 μM for 24 h. Levels of total CDC6 **(D)** and indicated chromatin bound proteins **(E)** were measured by Western Blot. DNA replication was determined by EdU incorporation assays **(F)**, and DNA damage was evaluated by alkaline comet assays **(G)** or immunofluorescence staining of γH2A.X **(H)**. **p < 0.01.

Deficiency in DNA replication licensing generates DNA damage, activates DNA damage response and ultimately induces death of cells (Burhans et al., 2002). Given the important roles of pre-replication complexes (pre-RCs) in the initiation of DNA replication, we next measured the effects of wogonin on assembly of pre-RCs onto chromatin. Chromatin-bounding assays showed that chromatin-bound pre-RC proteins including CDC6, MCM2, MCM3. MCM4, and MCM6 were significantly reduced in wogonin treated cells (**Figure 3A**). Moreover, the decrease of chromatin-bound pre-RC proteins was accompanied by a reduced loading of PCNA, a key factor necessary for DNA polymerase δ function (**Figure 3A**). Similarly, the total protein levels of MCM2, MCM6, and CDC6 were also decreased in wogonin treated cells (**Figure 3B**).

Binding of CDC6 protein to chromatin is critical for loading of MCM proteins and therefore for initiation of DNA replication (Cook et al., 2002). Depletion of CDC6 perturbs DNA replication and induces apoptosis in cancer cell lines (Lau et al., 2006). Western blot analysis showed that wogonin reduced expression of CDC6 in a concentration-dependent way (**Figure 3C**). Then we determined whether the inhibitive effects of wogonin on RCC cells were mediated by down-regulation of CDC6. Western Blot showed that transfection of CDC6 expression plasmid significantly restored the reduced endogenous CDC6 caused by wogonin (**Figure 3D**). As shown in **Figures 3E, F**, expression of exogenous CDC6 partially restored chromatin-bound MCM2, MCM6, and PCNA levels and reversed the inhibitory effect of wogonin on DNA replication. In addition, the mean olive tail moment values and γH2A.X levels were also significantly decreased by introduction of exogenous CDC6 (**Figures 3G, H**). Collectively, these results indicate that CDC6 is a key target of wogonin in RCC cells.

### Down-Regulation of CDC6 Inhibits Proliferation, Migration, and Invasion of RCC Cells

**Figure 4:**
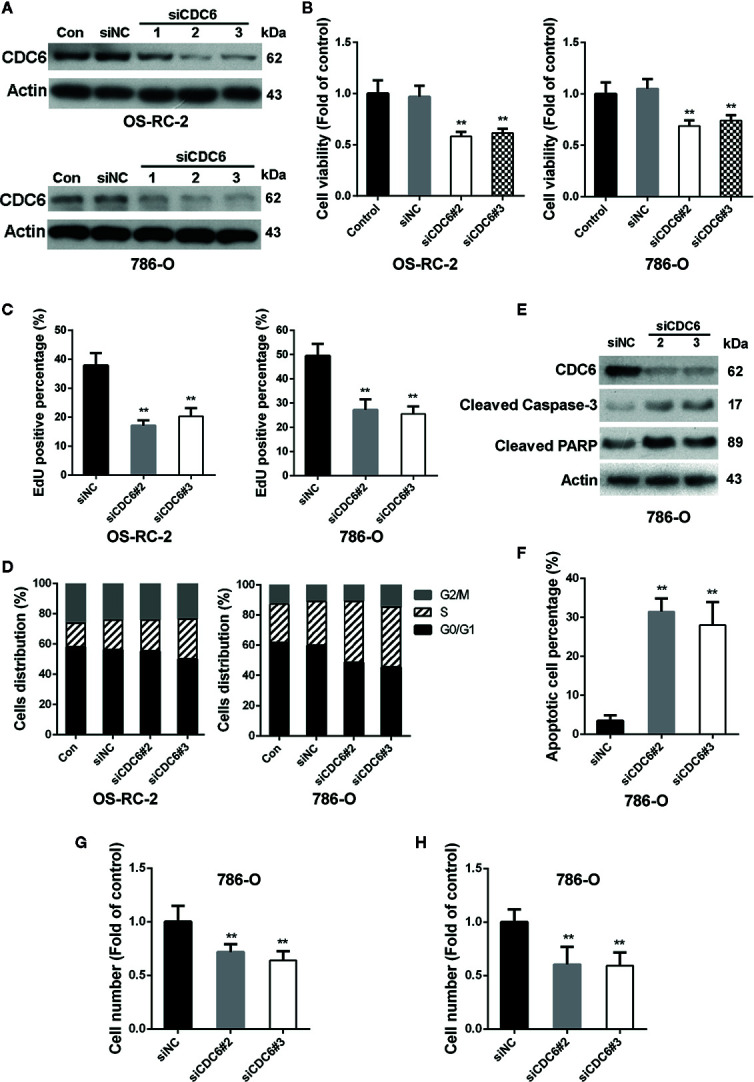
Down-regulation of CDC6 inhibits proliferation, migration and invasion of RCC cells. **(A**–**D)** Cells were transfected with indicated siRNAs for 72 h. Levels of CDC6 and cell viability were determined by Western Blot **(A)** and MTT assays **(B)**. **(C, D)** Cells were transfected with indicated siRNAs for 72 h. DNA replication and cell cycle distribution was determined by EdU incorporation assays **(C)** and flow cytometry **(D)**. **(E**, **F)** 786-O cells were transfected with siRNAs for 72 h. Cell apoptosis was then determined by Western Blot **(E)** and TUNEL staining **(F)**. **(G**, **H)** 786-O cells were transfected with siRNAs for 72 h, and ability of cell migration **(G)** and invasion **(H)** were evaluated by transwell assays. **p < 0.01.

CDC6 has been reported to be associated with the progression and prognosis in various cancers (Chen et al., 2016; Deng et al., 2016). To characterize the role of CDC6 in malignant properties of RCC cells, siRNAs targeting CDC6 were transfected into OS-RC-2 and 786-O cells (**Figure 4A**). MTT and EdU incorporation assays showed that down-regulation of CDC6 efficiently inhibited cell proliferation and DNA replication of both OS-RC-2 and 786-O cells (**Figures 4B, C**). We next determined whether CDC6 inhibition affected cell cycle progression of RCC cells. The results showed that knockdown of CDC6 could increase the proportion of cells in S phase (**Figure 4D** and **Supplementary Figure 3A**), indicating that inhibition of CDC6 induced S phase arrest in RCC cells.

Given the important role of CDC6 in DNA replication, we reasoned that depletion of CDC6 may induce apoptosis of RCC cells. Western blot analysis showed that expression of cleaved caspase-3 and cleaved PARP was significantly increased in CDC6 knockdown 786-O cells (**Figure 4E**). TUNEL assays also showed that depletion of CDC6 led to an increase in the percentage of apoptotic cells (**Figure 4F**).

Next, the impacts of CDC6 on migration and invasion of RCC cells were examined by transwell assay. The number of migrating and invading 786-O cells transfected with siCDC6 through the filter was apparently reduced (**Figures 4G, H**). Together, these results demonstrate that depletion of CDC6 inhibits proliferation, migration, invasion, and induces apoptosis of RCC cells.

To further explore the clinical significance of CDC6 in renal cell carcinoma, survival data from Oncolnc database (http://www.oncolnc.org) were used to determine the correlation between CDC6 expression and prognosis of RCC patients. As shown in **Supplementary Figure 3B**, renal clear cell patients with higher CDC6 expression had a poor prognosis than patients with lower CDC6 expression (P = 4.32e-07). Similarly, higher expression of CDC6 also predicted poor prognosis in patients with renal papillary cell carcinoma (P = 2.03e-05) (**Supplementary Figure 3C**).

### Wogonin Down-Regulates the Transcription of CDC6 *via* Inhibiting CDK4-RB Pathway

**Figure 5:**
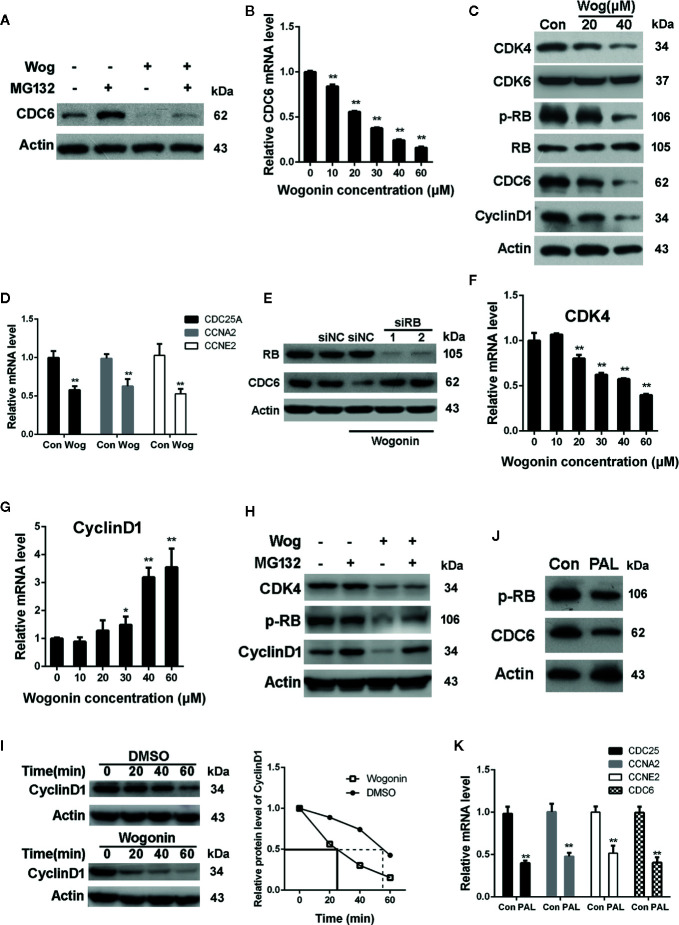
Wogonin downregulates the transcription of CDC6 *via* inhibiting CDK4-RB pathway. **(A)** 786-O cells were treated with 20 μM wogonin for 24 h and then MG132 for 4 h. Cells were harvested and levels of CDC6 were determined by Western blot. **(B)** 786-O cells were treated with different concentrations of wogonin for 24 h and mRNA levels of CDC6 were measured by qRT-PCR. **(C)** 786-O cells were treated with different concentrations of wogonin for 24 h and indicated protein levels were determined by Western blot. **(D)** qRT-PCR assays of indicated genes in 786-O cells treated with or without 20 μM wogonin for 24 h. **(E)** 786-O cells were transfected with indicated siRNA for 48 h and then 20 μM wogonin for 24 h. Levels of RB and CDC6 were determined by Western Blot. **(F**, **G)** qRT-PCR was performed to evaluate mRNA levels of CDK4 and Cyclin D1 in 786-O cells treated with different concentrations of wogonin for 24 h. **(H)** 786-O cells were treated with 20 μM wogonin for 24 h and then MG132 for 4 h. Cells were harvested and levels of CDK4, p-RB and Cyclin D1 were determined by Western blot. **(I)** 786-O cells were treated with DMSO or 20 μM wogonin for 24 h and then cycloheximide (100 μg/ml) for indicated time. Cells were harvested and levels of Cyclin D1 were determined by Western blot. **(J, K)** 786-O cells were treated with or without 20 μM palbociclib for 24 h, then protein levels of p-RB and CDC6 were measured by Western blot and levels of indicated mRNAs were measured by qRT-PCR. **p < 0.01.

We next examined the effects of wogonin on protein degradation of CDC6. As shown in **Figure 5A**, the level of CDC6 was mildly upregulated after MG132 treatment in wogonin treated cells, suggesting MG132 inhibited proteasomal-mediated degradation of the existing CDC6 protein. However, the CDC6 protein level was still significantly decreased compared to that of DMSO and MG132 treated cells, indicating that downregulation of CDC6 by wogonin is not due to proteasome-mediated degradation. Real-time PCR analysis showed that CDC6 mRNA was significantly decreased by wogonin in a concentration-dependent manner (**Figure 5B**), suggesting that wogonin may disturb the transcription of CDC6 in RCC cells.

It is well established that CDK4/6 combine with D-type cyclins to phosphorylate the retinoblastoma (RB) tumor suppressor, releasing E2F transcription factor, leading to the transcription of genes critical for the progression of S phase, including CDC6 (Braden et al., 2008). Thus we determined the activation of CDK4/6 pathway through analysis of phosphorylated RB (p-RB) protein levels. The results showed that wogonin treatment resulted in a marked decrease in the levels of p-RB, Cyclin D1, and CDK4 (**Figure 5C**). However, no parallel reduction in total RB and CDK6 expression were observed (**Figure 5C**). Similar with the changes of p-RB, mRNA levels of CDC25A, CCNE2, and CCNA2, known as the downstream targets of CDK4-RB, were also decreased in wogonin treated cells (**Figure 5D**). These data suggest that wogonin impairs CDK4 activity in RCC cells. In order to confirm that down-regulation of CDC6 by wogonin is through inhibiting CDK4-RB pathway, siRNA was used to knockdown the expression of RB. The results showed that RNAi of RB blocked the decrease of CDC6 induced by wogonin (**Figure 5E**).

To further characterize the mechanisms that wogonin inhibit CDK4 activity, expression of CDK4 and Cyclin D1 were determined. Consistent with the decreased protein levels of CDK4, treatment of wogonin also significantly reduced CDK4 mRNA levels in a concentration-dependent manner (**Figure 5F**). However, no corresponding change of Cyclin D1 mRNA was observed (**Figure 5G**). Thus we further determined whether wogonin may promote the degradation of Cyclin D1 protein. As shown in **Figure 5H**, MG132 blocked the reduction of Cyclin D1 induced by wogonin. Moreover, cycloheximide (CHX) was also used to explore the effects of wogonin on the half-life of Cyclin D1 and the results showed an obvious reduced half-life of Cyclin D1 induced by wogonin (**Figure 5I**), suggesting that wogonin accelerates proteasomal degradation of Cyclin D1. Importantly, similar with that of wogonin, palbociclib also significantly reduced protein levels of p-RB and CDC6 (**Figure 5J**) as well as mRNA levels of CDC25A, CCNE2, CCNA2, and CDC6 (**Figure 5K**). Collectively, these results suggest that wogonin down-regulates CDC6 expression through inhibition of CDK4-RB pathway.

### Wogonin Reverses Resistance to Sunitinib in RCC Cells *via* Inhibition of CDK4-RB Pathway

**Figure 6:**
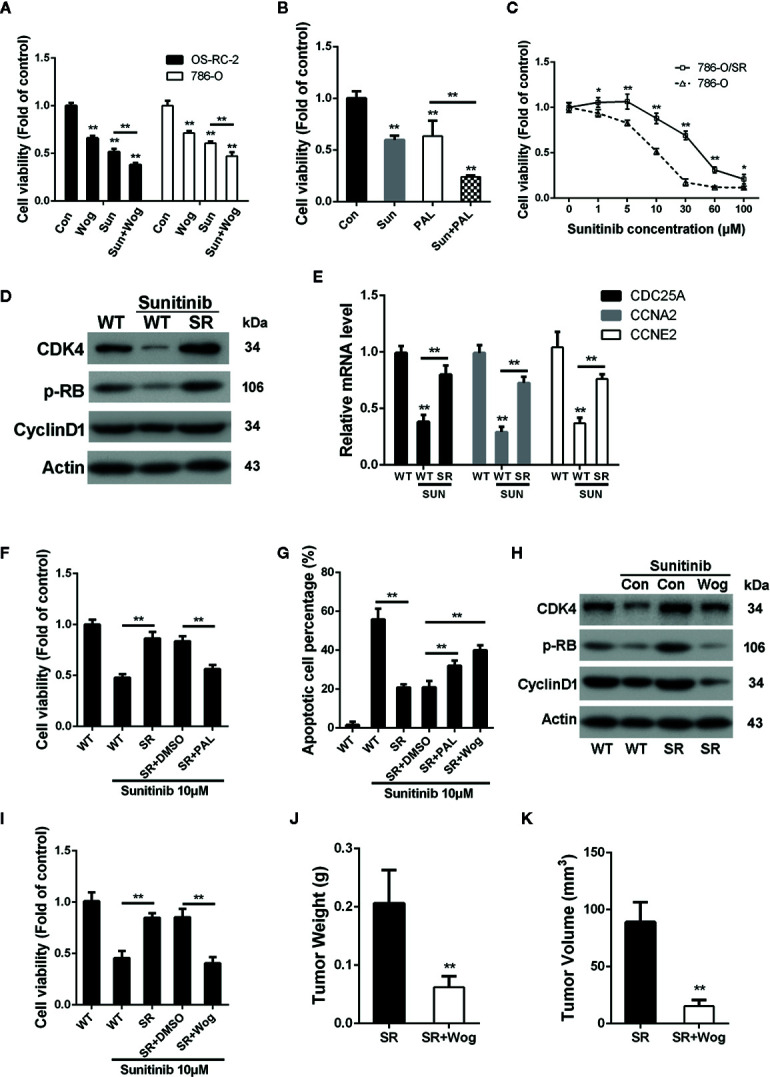
Wogonin overcomes resistance of RCC cells to sunitinib through inhibiting CDK4-RB pathway. **(A)** Cells were treated with or without 40 μM wogonin or 10 μM sunitinib (Sun) alone or both agents for 24 h, and cell viability was measured by MTT assays. **(B)** 786-O cells were treated with or without 20 μM pabociclib or 10 μM sunitinib alone or both agents for 24 h, and cell viability was measured by MTT assays. **(C)** Wildtype (WT) or sunitinib-resistant (SR) 786-O cells were treated with different concentrations of sunitinib for 48 h, and cell viability was measured by MTT assays. **(D)** Protein levels in indicated cells treated with or without sunitinib at 10 μM for 24 h were determined by Western blot. **(E)** qRT-PCR assays of indicated genes in 786-O wildtype (WT) or sunitinib-resistant (SR) cells treated with or without 10 μM sunitinib for 24 h. **(F–I)** Indicated cells were treated with 10 μM sunitinib alone or together with 20 μM palbociclib or 20 μM wogonin for 24 h. Cell viability and apoptosis were determined by MTT assays **(F, I)** and TUNEL assays **(G)**. Indicated protein levels were determined by western blot **(H)**. **(J, K)** 1×10^6^ 786-O/SR cells were injected subcutaneously into nude mice. Mice were either treated with wogonin (40 mg/kg) and sunitinib (20 mg/kg) or treated with sunitinib (20 mg/kg) alone (as control) everyday for 2 weeks. Then the mice were sacrificed, and the tumor weight **(J)** and tumor volume **(K)** were measured. *p < 0.05; **p < 0.01.

Sunitinib, a multi-target RTK inhibitor, has become the mainstay of therapies for advanced RCC patients (Oudard et al., 2011). We further determined whether wogonin could sensitize RCC cells to sunitinib. The results showed that combined treatment of wogonin and sunitinib demonstrated significantly stronger inhibitive effects than sunitinib alone (**Figure 6A**). The finding that wogonin suppressed CDK4 activity prompted us to further determine the effect of palbociclib on inhibition of cell viability induced by sunitinib in RCC cells. Similarly, palbocilib also enhanced inhibitive efficacy of sunitinib in 786-O cells (**Figure 6B**).

Despite most RCC patients could benefit from sunitinib treatment, a number of patients demonstrated drug resistance and disease progression after receiving sunitinib treatment for 6-15 months. As studies have reported the important role of CDK4 signal pathway in the development of resistance to target therapy in other malignancies (Yadav et al., 2014; Goel et al., 2016), we next examined the roles of CDK4-RB pathway in sunitinib resistance in RCC cells.

As shown in **Figure 6C**, sunitinib resistant 786-O cells (786-O/SR) exhibited a significantly increased cell viability compared with wildtype 786-O cells when treated with sunitinib. Western Blot showed that levels of p-RB were reduced by sunitinib treatment in wildtype 786-O cells (**Figure 6D**). In contrast, 786-O/SR cells demonstrated a significantly higher level of p-RB and CDK4 (**Figure 6D**). Furthermore, an increased expression of the mRNA levels of CDC25A, CCNE2, and CCNA2 were also observed in 786-O/SR cells (**Figure 6E**). Similarly, significantly elevated levels of p-RB, CDK4, and Cyclin D1 were also observed in OS-RC-2/SR and TK-10/SR cells (**Supplementary Figures 4A–D**), indicating that CDK4-RB pathway was reactivated in sunitinib resistant RCC cells.

We further explored whether the activated CDK4-RB was involved in sunitinib resistance. The results of MTT assays showed that palbociclib significantly decreased cell viability of 786-O/SR cells treated with sunitinib (**Figure 6F**). Moreover, the percentage of apoptotic cells was also increased by palbociclib treatment (**Figure 6G**). These results indicate that resistance of RCC to sunitinib may be associated with activation of CDK4-RB pathway, and inhibition of CDK4 could restore sensitivity of sunitinib resistant RCC cells.

As wogonin inhibits CDK4 in RCC cells, we next asked whether wogonin could also reverse sunitinib resistance. Western blot showed that wogonin significantly decreased the levels of CDK4, Cyclin D1 and p-RB in 786-O/SR and OS-RC-2/SR cells (**Figure 6H** and **Supplementary Figure 4B**). Moreover, wogonin could also efficiently induces apoptosis and decreases cell viability of 786-O/SR cells (**Figures 6G, I**).

The results of *in vivo* experiments demonstrated that application of wogonin and sunitinib significantly suppressed tumor growth after subcutaneous injecting of 786-O/SR cells (**Supplementary Figure 4E**). The average tumor weight and volume were significantly reduced in the group receiving wogonin and sunitinib compared with sunitinib alone (**Figures 6J, K**). Moreover, administration of wogonin and sunitinib also inhibited the expression of CDK4, p-RB, and Cyclin D1 in tumor tissues (**Supplementary Figure 4F**).

## Discussion

Treatment of RCC has undergone a great change during the last decade. Agents targeting VEGF receptors or mTOR, such as sunitinib and everolimus, are commonly used in mRCC patients (Jonasch et al., 2014). However, most patients ultimately underwent cancer progression. Therefore, new drugs for RCC treatment are highly desirable. In the present study, we demonstrated that wogonin significantly inhibited proliferation, migration, and invasion of RCC cells. In addition, we found that wogonin induced DNA damage, G2/M phase arrest, and apoptosis of RCC cells. Importantly, wogonin exhibited much less evident anti-proliferation effects on normal human embryonic kidney HEK-293 cells. These findings indicate that wogonin may possess selective cytotoxic effects on RCC cells.

CDC6 is able to facilitate loading of mini-chromosome maintenance (MCM) complex on chromatin and assemble pre-RC at origins to initiate DNA replication (Kelly and Brown, 2000). CDC6 also inhibits microtubule organizing activity of the centrosome (Lee et al., 2017), which functions in cell migration(Kushner et al., 2014) and signal transduction (Watanabe et al., 2005). Previous studies reported the important roles of CDC6 in different malignancies, such as hepatocellular carcinoma (Xiong et al., 2008) and prostate cancer (Wu et al., 2009). High expression of CDC6 is associated with accelerated proliferation and poor prognosis (Deng et al., 2016). CDC6 has also been reported to be associated with cisplatin resistance by activation of ATR-Chk1 pathway in bladder cancer cells (Chen et al., 2016). Here, we found that silencing CDC6 significantly inhibited proliferation, DNA replication, migration, and invasion of RCC cells, resulting in a S phase arrest and cell apoptosis. Data from Oncolnc showed that the expression of CDC6 was related to the prognosis of RCC patients. High expression of CDC6 predicted poor survival rate of patients with renal clear cell carcinoma and renal papillary cell carcinoma, suggesting that CDC6 may serve as a potential target for RCC management. In addition, we showed that expression of CDC6 was down-regulated by wogonin treatment. More importantly, introduction of exogenous CDC6 effectively reversed the inhibitory effects of wogonin on DNA replication and DNA damage in RCC cells, indicating that CDC6 is a target of wogonin in RCC cells. However, as a natural product, wogonin may have multi-targets in RCC cell. Our results showed that wogonin inhibited CDK4, the downstream of which is CDC6. Therefore, as only one of the targets through which wogonin regulated the proliferation of RCC cells, simply restored CDC6 would not be able to completely reverse the inhibitory effects of wogonin in RCC cells. Similarly, besides CDC6, wogonin has other targets, which may be involved in G2/M phase transition. Therefore, though knockdown of CDC6 caused S phase arrest, wogonin treatment resulted in G2/M phase arrest. Researches have reported the role of CDC6 in EMT. CDC6 could regulate the expression of E- and N-Cadherin to affect the migration and invasion of trophoblast cells in preeclampsia (Yang and Meng, 2020). Targeting CDC6 has also been shown to sensitized cancer cells to ionizing radiation-induced apoptosis, and reversed EMT (Yu et al., 2019). Consistently, our results demonstrated that wogonin inhibited EMT of RCC cells, suggesting that wogonin may decrease the migration and invasion *via* targeting CDC6 to suppress EMT.

Increasing evidence have recognized CDC6 as a critical factor in DNA damage response. CDC6 could physically interact with ATR which is enhanced by CDC6 phosphorylation by CDK, and CDC6 silencing impaired ATR-dependent replication-checkpoint response (Yoshida et al., 2010). Targeting CDC6 together with Chk1/2 inhibitor could suppress TopBP1-ATR-Chk1 signaling and increase phosphorylation of ATM, a biomarker of DNA damage, synergistically increasing the treatment efficacy in prostate cancer cells (Karanika et al., 2017). Furthermore, ATR-mediated phosphorylation of CDC6 is also critical for the maintenance of high chromatin levels of CDC6 protein (Liu et al., 2009). Our results showed that wogonin induced elevation of genomic instability of RCC cell (**Figure 2E**), indicating that wogonin might affect the genomic stability of RCC cells through inhibiting CDC6 expression, leading to inactivation of ATR-Chk1 pathway and resulting in the DNA damage and apoptosis of RCC cells.

Our results showed that wogonin inhibited the transcription of CDC6. In G1 phase, CDK4/6 combines with D- type cyclins to phosphorylate RB, allowing for releasing E2F and subsequent transcription of E2F target genes including CDC6 (Sherr, 2000). We found that the protein levels of CDK4 as well as p-RB and Cyclin D1 were down-regulated by wogonin in RCC cells, suggesting that wogonin may exert anti-cancer efficacy by suppressing CDK4 and Cyclin D1 expression. Disruption of CDK4/6-RB pathway and an uncontrolled G1 to S transition is a common feature of cancer cells (Hamilton and Infante, 2016). Therefore, CDK4/6 have long been regarded as promising targets for cancer therapies. Clinical studies have demonstrated that addition of selective CDK4/6 inhibitor palbociclib to letrozole or fulvestrant showed an improvement in median progression-free survival (PFS) in patients with HR-positive advanced-stage breast cancer (Finn et al., 2015; Cristofanilli et al., 2016). Preclinical evidence of effectiveness of CDK4/6 inhibition also exists in other malignancies, such as myeloma (Niesvizky et al., 2015) and melanoma (Kwong et al., 2012). However, a report showed that palbociclib treatment specifically stabilized activated cyclin D3-CDK4/6 complexes that were devoid of p21 and p27, paradoxical inducing RB phosphorylation, DNA replication and cell cycle entry in the absence of a mitogenic stimulation (Paternot et al., 2014). Thus inhibiting expression of CDK4/6 proteins may be more effective than simply suppressing its activity in treatment of cancer. Cyclin D1 plays an important role in the development and progression of several cancers including breast cancer and bladder cancer (Knudsen et al., 2006; Musgrove, 2006). Induction of Cyclin D1 degradation is regarded to be a promising avenue for therapeutic intervention of cancer. Our results that wogonin inhibited the transcription of CDK4 and accelerated the protein degradation of CyclinD1 indicated that wogonin may be a promising drug in the future treatment of RCC.

Sunitinib, known as PDGFR and VEGFR inhibitor, could significantly increase the PFS and overall survival (OS) in patients with mRCC (Motzer et al., 2007). Here, we found that combination of wogonin and sunitinib demonstrated stronger inhibitory efficiency than sunitinib alone in OS-RC-2 and 786-O cells. Moreover, we also showed that wogonin effectively reversed resistance to sunitinib in RCC cells. Though a large number of patients with mRCC could benefit from sunitinib treatment, most of them eventually develop drug resistance. Up-regulation of proangiogenic pathways and changes of tumor microenvironment have been considered to be important reasons for TKIs resistance (Joosten et al., 2015). IL-8 was reported to be a contributor to sunitinib resistance in RCC and may serve as a candidate therapeutic target to reverse sunitinib resistance (Huang et al., 2010a). Moreover, inhibition of AXL and MET activity or addition of MEK inhibitor was also reported to abrogate the resistance to sunitinib (Diaz-Montero et al., 2016; Zhou et al., 2016). Here, we found that levels of CDK4 and p-RB were reduced in sunitinib treated wild-type 786-O (786-O/WT) and OS-RC-2 (OS-RC-2/WT) cells. Interestingly, much higher levels of CDK4 as well as Cyclin D1 and p-RB were observed in the 786-O/SR, OS-RC-2/SR, and TK-10/SR cells, indicating the activation of CDK4-RB pathway. More importantly, we found that application of CDK4/6 inhibitor palbociclib effectively restored the sensitivity of sunitinib in 786-O/SR cells. Based on these findings, hyperactivation of CDK4-RB may confer to the resistance to sunitinib in RCC. To our knowledge, this is the first study to demonstrate a significant correlation between CDK4-RB pathway activation and sunitinib resistance. A previous study revealed that targeting CDK4/6 could overcome MAPK-mediated resistance to B-RAF inhibitors in B-RAF V600E melanoma (Yadav et al., 2014). Moreover, Shom Goe et al. reported that CDK4 mediated the resistance to targeted therapy in HER2-positive breast cancer and can be reversed by CDK4/6 inhibitor (Goel et al., 2016). Our discovery that resistance to sunitinib in RCC cells is associated with hyperactivation of CDK4-RB suggests CDK4 as a potential target in future treatment of mRCC patients who have developed resistance to sunitinib.

In summary, this study demonstrated that wogonin could efficiently inhibit cell proliferation, migration, invasion, and induce apoptosis of RCC cells. Moreover, we also showed that the cytotoxic effects of wogonin were achieved through suppression of CDK4-RB pathway and subsequently, down-regulating CDC6. Furthermore, this study indicated that hyperactivation of CDK4 was a possible mechanism of sunitinib resistance in RCC cells, which can be reversed by wogonin. Considering low toxicity, rapid absorption, and long residence time after oral administration (Cai et al., 2016), wogonin may be an attractive candidate for treatment of RCC patients.

## Data Availability Statement

The original contributions presented in the study are included in the article/**Supplementary Material**; further inquiries can be directed to the corresponding authors.

## Ethics Statement

The animal study was reviewed and approved by Ethics Committee of Qilu Hospital of Shandong University.

## Author Contributions

YZo, BS, YW, and SC contributed to the conception of the study. YW, SC, SS, GL, LC, YX, and JC contributed to experimental technology and experimental design. WW, XJ, LZ, and YZh performed the data analyses and animal experiment guide. YZo, BS, YW, and SC wrote the manuscript.

## Funding

This work was supported by the National Natural Science Foundation of China (grants 31371369 and 31671427 to YZo, grant 81804104 to SS, grants 81470987 and 81670687 to BS, grant 31570912 to LZ, grant 81770660 to GL), the Tai Shan Scholar Foundation to BS, the Key Research and Development Plan of Shandong Province (2015GSF118029 to YZo) and Young Scholars Program of Shandong University (to YZo), the Science and Technology Development Project of Jinan (grant 201602155 to BS), Natural Science Foundation of Shandong Province (grant ZR2014CQ006 to LZ, grant ZR2014HQ062 to YZh), Primary Research and Development Plan of Shandong Province (grant 2016GSF201036 to YZh).

## Conflict of Interest

The authors declare that the research was conducted in the absence of any commercial or financial relationships that could be construed as a potential conflict of interest.

## Supplementary Material

Figure S1 Wogonin inhibits proliferation, migration and invasion of RCC cells. **A**. Colony formation efficiency of OS-RC-2 and 786-O cells treated with or without wogonin. **B.** EdU incorporation assays of OS-RC-2 and 786-O cells treated with or without different concentrations of wogonin for 24 h (scale bar, 100 μm). **C.** MTT assays of HEK-293 cells treated with different concentrations of wogonin for 48 h. **D.** Colony formation efficiency of HEK-293 cells treated with or without wogonin. **E.** Tumor formation by 786-O cells in nude mice. 1×106 786-O cells were injected subcutaneously into nude mice. Mice were either treated with wogonin (40 mg/kg) or treated with vehicle (as control) everyday for 2 weeks. **F–G.** Cells were treated with different concentrations of wogonin for 24 h, and transwell assays were performed to evaluate the ability of cell migration (F) and invasion (G) (scale bar, 50 μm).

Figure S2 Wogonin induces apoptosis of RCC cells. **A.** OS-RC-2 and 786-O cells were treated with or without different concentrations of wogonin for 24 h and cell cycle distribution was determined by flow cytometry. **B.** TUNEL assays of 786-O cells treated with or without wogonin at different concentrations for 24h (scale bar, 50 μm). **C.** OS-RC-2 and 786-O cells were treated with or without different concentrations of wogonin for 48 h and SA-β-gal activity assays were performed.

Figure S3 CDC6 expression is correlated with cell cycle of RCC cells and prognosis of RCC patients. **A.** Cells were transfected with indicated siRNAs for 72 h. Cell cycle distribution was determined by flow cytometry. **B.** Kaplan–Meier survival analysis for CDC6 expression in renal clear cell carcinoma patients from Oncolnc. **C.** Kaplan–Meier survival analysis for CDC6 expression in renal papillary cell carcinoma patients from Oncolnc.

Figure S4 Wogonin suppresses CDK4-RB pathway in sunitinib resistant RCC cells and inhibits proliferation of 786-O/SR cells in vivo. **A.** Wildtype (WT) or sunitinib-resistant (SR) OS-RC-2 cells were treated with different concentrations of sunitinib for 48 h and cell viability was measured by MTT assays. **B.** OS-RC-2 or OS-RC/SR cells were treated with 10 μM sunitinib alone or together 40 μM wogonin for 24 h. Indicated protein levels were determined by Western blot. **C.** Wildtype (WT) or sunitinib-resistant (SR) TK-10 cells were treated with different concentrations of sunitinib for 48 h and cell viability was measured by MTT assays. **D.** The protein levels of CDK4, p-RB and Cylcin D1 were determined by Western blot in TK-10/WT and TK-10/SR cells after being treated with 10 μM sunitinib for 24 h. **E.** Tumor formation by 786-O/SR cells in nude mice. 1×106 786-O/SR cells were injected subcutaneously into nude mice. Mice were either treated with wogonin (40 mg/kg) and sunitinib (20 mg/kg) or treated with sunitinib (20 mg/kg) alone (as control) everyday for 2 weeks. **F.** The expression of CDK4, p-RB, CyclinD1 and CDC6 in tumor tissues were determined by Western blot.

## References

[B1] Baumann S. Fas S. C. Giaisi M. Muller W. W. Merling A. Gulow K. (2008). Wogonin preferentially kills malignant lymphocytes and suppresses T-cell tumor growth by inducing PLCgamma1- and Ca2+-dependent apoptosis. Blood 111, 2354–2363. 10.1182/blood-2007-06-096198 18070986

[B2] Braden W. A. Mcclendon A. K. Knudsen E. S. (2008). Cyclin-dependent kinase 4/6 activity is a critical determinant of pre-replication complex assembly. Oncogene 27, 7083–7093. 10.1038/onc.2008.319 18776921

[B3] Buczek M. Escudier B. Bartnik E. Szczylik C. Czarnecka A. (2014). Resistance to tyrosine kinase inhibitors in clear cell renal cell carcinoma: from the patient’s bed to molecular mechanisms. Biochim. Biophys. Acta 1845, 31–41. 10.1016/j.bbcan.2013.10.001 24135488

[B4] Burhans W. C. Blanchard F. Baumann H. (2002). Origin licensing and programmed cell death: a hypothesis. Cell Death Differ. 9, 870–872. 10.1038/sj.cdd.4401086 12181737

[B5] Cai Y. Li S. Li T. Zhou R. Wai A. T. Yan R. (2016). Oral pharmacokinetics of baicalin, wogonoside, oroxylin A 7-O-beta-d-glucuronide and their aglycones from an aqueous extract of Scutellariae Radix in the rat. J. Chromatogr. B. Analyt. Technol. BioMed. Life Sci. 1026, 124–133. 10.1016/j.jchromb.2015.11.049 26809374

[B6] Chen S. Chen X. Xie G. He Y. Yan D. Zheng D. (2016). Cdc6 contributes to cisplatin-resistance by activation of ATR-Chk1 pathway in bladder cancer cells. Oncotarget 7, 40362–40376. 10.18632/oncotarget.9616 PMC5130013 27246979

[B7] Cook J. G. Park C. H. Burke T. W. Leone G. Degregori J. Engel A. (2002). Analysis of Cdc6 function in the assembly of mammalian prereplication complexes. Proc. Natl. Acad. Sci. U.S.A. 99, 1347–1352. 10.1073/pnas.032677499 11805305 PMC122193

[B8] Cristofanilli M. Turner N. C. Bondarenko I. Ro J. Im S. A. Masuda N. (2016). Fulvestrant plus palbociclib versus fulvestrant plus placebo for treatment of hormone-receptor-positive, HER2-negative metastatic breast cancer that progressed on previous endocrine therapy (PALOMA-3): final analysis of the multicentre, double-blind, phase 3 randomised controlled trial. Lancet Oncol. 17, 425–439. 10.1016/S1470-2045(15)00613-0 26947331

[B9] Deng Y. Jiang L. Wang Y. Xi Q. Zhong J. Liu J. (2016). High expression of CDC6 is associated with accelerated cell proliferation and poor prognosis of epithelial ovarian cancer. Pathol. Res. Pract. 212, 239–246. 10.1016/j.prp.2015.09.014 26920249

[B10] Diaz-Montero C. M. Mao F. J. Barnard J. Parker Y. Zamanian-Daryoush M. Pink J. J. (2016). MEK inhibition abrogates sunitinib resistance in a renal cell carcinoma patient-derived xenograft model. Br. J. Cancer 115, 920–928. 10.1038/bjc.2016.263 27560553 PMC5061902

[B11] Finn R. S. Crown J. P. Lang I. Boer K. Bondarenko I. M. Kulyk S. O. (2015). The cyclin-dependent kinase 4/6 inhibitor palbociclib in combination with letrozole versus letrozole alone as first-line treatment of oestrogen receptor-positive, HER2-negative, advanced breast cancer (PALOMA-1/TRIO-18): a randomised phase 2 study. Lancet Oncol. 16, 25–35. 10.1016/S1470-2045(14)71159-3 25524798

[B12] Goel S. Wang Q. Watt A. C. Tolaney S. M. Dillon D. A. Li W. (2016). Overcoming Therapeutic Resistance in HER2-Positive Breast Cancers with CDK4/6 Inhibitors. Cancer Cell 29, 255–269. 10.1016/j.ccell.2016.02.006 26977878 PMC4794996

[B13] Hamilton E. Infante J. R. (2016). Targeting CDK4/6 in patients with cancer. Cancer Treat Rev. 45, 129–138. 10.1016/j.ctrv.2016.03.002 27017286

[B14] He L. Lu N. Dai Q. Zhao Y. Zhao L. Wang H. (2013). Wogonin induced G1 cell cycle arrest by regulating Wnt/beta-catenin signaling pathway and inactivating CDK8 in human colorectal cancer carcinoma cells. Toxicology 312, 36–47. 10.1016/j.tox.2013.07.013 23907061

[B15] Hu C. Xu M. Qin R. Chen W. Xu X. (2015). Wogonin induces apoptosis and endoplasmic reticulum stress in HL-60 leukemia cells through inhibition of the PI3K-AKT signaling pathway. Oncol. Rep. 33, 3146–3154. 10.3892/or.2015.3896 25846394

[B16] Huang D. Ding Y. Zhou M. Rini B. I. Petillo D. Qian C. N. (2010a). Interleukin-8 mediates resistance to antiangiogenic agent sunitinib in renal cell carcinoma. Cancer Res. 70, 1063–1071. 10.1158/0008-5472.CAN-09-3965 20103651 PMC3719378

[B17] Huang S. T. Wang C. Y. Yang R. C. Chu C. J. Wu H. T. Pang J. H. (2010b). Wogonin, an active compound in Scutellaria baicalensis, induces apoptosis and reduces telomerase activity in the HL-60 leukemia cells. Phytomedicine 17, 47–54. 10.1016/j.phymed.2009.06.005 19577445

[B18] Jonasch E. Gao J. Rathmell W. K. (2014). Renal cell carcinoma. BMJ 349, g4797. 10.1136/bmj.g4797 25385470 PMC4707715

[B19] Joosten S. C. Hamming L. Soetekouw P. M. Aarts M. J. Veeck J. Van Engeland M. (2015). Resistance to sunitinib in renal cell carcinoma: From molecular mechanisms to predictive markers and future perspectives. Biochim. Biophys. Acta 1855, 1–16. 10.1016/j.bbcan.2014.11.002 25446042

[B20] Karanika S. Karantanos T. Li L. Wang J. Park S. Yang G. (2017). Targeting DNA Damage Response in Prostate Cancer by Inhibiting Androgen Receptor-CDC6-ATR-Chk1 Signaling. Cell Rep. 18, 1970–1981. 10.1016/j.celrep.2017.01.072 28228262 PMC5349188

[B21] Kelly T. J. Brown G. W. (2000). Regulation of chromosome replication. Annu. Rev. Biochem. 69, 829–880. 10.1146/annurev.biochem.69.1.829 10966477

[B22] Kim E. H. Jang H. Shin D. Baek S. H. Roh J. L. (2016). Targeting Nrf2 with wogonin overcomes cisplatin resistance in head and neck cancer. Apoptosis 21, 1265–1278. 10.1007/s10495-016-1284-8 27544755

[B23] Knudsen K. E. Diehl J. A. Haiman C. A. Knudsen E. S. (2006). Cyclin D1: polymorphism, aberrant splicing and cancer risk. Oncogene 25, 1620–1628. 10.1038/sj.onc.1209371 16550162

[B24] Kushner E. J. Ferro L. S. Liu J. Y. Durrant J. R. Rogers S. L. Dudley A. C. (2014). Excess centrosomes disrupt endothelial cell migration via centrosome scattering. J. Cell Biol. 206, 257–272. 10.1083/jcb.201311013 25049273 PMC4107782

[B25] Kwong L. N. Costello J. C. Liu H. Jiang S. Helms T. L. Langsdorf A. E. (2012). Oncogenic NRAS signaling differentially regulates survival and proliferation in melanoma. Nat. Med. 18, 1503–1510. 10.1038/nm.2941 22983396 PMC3777533

[B26] Lau E. Zhu C. Abraham R. T. Jiang W. (2006). The functional role of Cdc6 in S-G2/M in mammalian cells. EMBO Rep. 7, 425–430. 10.1038/sj.embor.7400624 16439999 PMC1456921

[B27] Lee D. H. Lee T. H. Jung C. H. Kim Y. H. (2012). Wogonin induces apoptosis by activating the AMPK and p53 signaling pathways in human glioblastoma cells. Cell Signal 24, 2216–2225. 10.1016/j.cellsig.2012.07.019 22846543

[B28] Lee I. Kim G. S. Bae J. S. Kim J. Rhee K. Hwang D. S. (2017). The DNA replication protein Cdc6 inhibits the microtubule-organizing activity of the centrosome. J. Biol. Chem. 292, 16267–16276. 10.1074/jbc.M116.763680 28827311 PMC5625056

[B29] Liu L. Choi J. H. Yim H. Choi J. S. Park B. D. Cho S. J. (2009). ATR (AT mutated Rad3 related) activity stabilizes Cdc6 and delays G2/M-phase entry during hydroxyurea-induced S-phase arrest of HeLa cells. Int. J. Biochem. Cell Biol. 41, 1410–1420. 10.1016/j.biocel.2008.12.014 19154794

[B30] Liu X. Tian S. Liu M. Jian L. Zhao L. (2016). Wogonin inhibits the proliferation and invasion, and induces the apoptosis of HepG2 and Bel7402 HCC cells through NFkappaB/Bcl-2, EGFR and EGFR downstream ERK/AKT signaling. Int. J. Mol. Med. 38, 1250–1256. 10.3892/ijmm.2016.2700 27499272

[B31] Ljungberg B. Bensalah K. Canfield S. Dabestani S. Hofmann F. Hora M. (2015). EAU guidelines on renal cell carcinoma: 2014 update. Eur. Urol. 67, 913–924. 10.1016/j.eururo.2015.01.005 25616710

[B32] Lu H. Gao F. Shu G. Xia G. Shao Z. Lu H. (2015). Wogonin inhibits the proliferation of myelodysplastic syndrome cells through the induction of cell cycle arrest and apoptosis. Mol. Med. Rep. 12, 7285–7292. 10.3892/mmr.2015.4353 26398525 PMC4626188

[B33] Mailand N. Diffley J. F. (2005). CDKs promote DNA replication origin licensing in human cells by protecting Cdc6 from APC/C-dependent proteolysis. Cell 122, 915–926. 10.1016/j.cell.2005.08.013 16153703

[B34] Mi J. Zou Y. Lin X. Lu J. Liu X. Zhao H. (2017). Dysregulation of the miR-194-CUL4B negative feedback loop drives tumorigenesis in non-small-cell lung carcinoma. Mol. Oncol. 11, 305–319. 10.1002/1878-0261.12038 28164432 PMC5527444

[B35] Motzer R. J. Hutson T. E. Tomczak P. Michaelson M. D. Bukowski R. M. Rixe O. (2007). Sunitinib versus interferon alfa in metastatic renal-cell carcinoma. N. Engl. J. Med. 356, 115–124. 10.1056/NEJMoa065044 17215529

[B36] Musgrove E. A. (2006). Cyclins: roles in mitogenic signaling and oncogenic transformation. Growth Factors 24, 13–19. 10.1080/08977190500361812 16393691

[B37] Niesvizky R. Badros A. Z. Costa L. J. Ely S. A. Singhal S. B. Stadtmauer E. A. (2015). Phase 1/2 study of cyclin-dependent kinase (CDK)4/6 inhibitor palbociclib (PD-0332991) with bortezomib and dexamethasone in relapsed/refractory multiple myeloma. Leuk Lymphoma 56, 3320–3328. 10.3109/10428194.2015.1030641 25813205

[B38] Norbury C. J. Zhivotovsky B. (2004). DNA damage-induced apoptosis. Oncogene 23, 2797–2808. 10.1038/sj.onc.1207532 15077143

[B39] Oudard S. Beuselinck B. Decoene J. Albers P. (2011). Sunitinib for the treatment of metastatic renal cell carcinoma. Cancer Treat Rev. 37, 178–184. 10.1016/j.ctrv.2010.08.005 20817406

[B40] Paternot S. Colleoni B. Bisteau X. Roger P. P. (2014). The CDK4/CDK6 inhibitor PD0332991 paradoxically stabilizes activated cyclin D3-CDK4/6 complexes. Cell Cycle 13, 2879–2888. 10.4161/15384101.2014.946841 25486476 PMC4615110

[B41] Qian C. Wang Y. Zhong Y. Tang J. Zhang J. Li Z. (2014). Wogonin-enhanced reactive oxygen species-induced apoptosis and potentiated cytotoxic effects of chemotherapeutic agents by suppression Nrf2-mediated signaling in HepG2 cells. Free Radic. Res. 48, 607–621. 10.3109/10715762.2014.897342 24666416

[B42] Sherr C. J. (2000). Cell cycle control and cancer. Harvey Lect. 96, 73–92. 12200872

[B43] Watanabe T. Noritake J. Kaibuchi K. (2005). Regulation of microtubules in cell migration. Trends Cell Biol. 15, 76–83. 10.1016/j.tcb.2004.12.006 15695094

[B44] Wu Z. Cho H. Hampton G. M. Theodorescu D. (2009). Cdc6 and cyclin E2 are PTEN-regulated genes associated with human prostate cancer metastasis. Neoplasia 11, 66–76. 10.1593/neo.81048 19107233 PMC2606120

[B45] Xiong X. D. Fang J. H. Qiu F. E. Zhao J. Cheng J. Yuan Y. (2008). A novel functional polymorphism in the Cdc6 promoter is associated with the risk for hepatocellular carcinoma. Mutat. Res. 643, 70–74. 10.1016/j.mrfmmm.2008.06.006 18640135

[B46] Yadav V. Burke T. F. Huber L. Van Horn R. D. Zhang Y. Buchanan S. G. (2014). The CDK4/6 inhibitor LY2835219 overcomes vemurafenib resistance resulting from MAPK reactivation and cyclin D1 upregulation. Mol. Cancer Ther. 13, 2253–2263. 10.1158/1535-7163.MCT-14-0257 25122067

[B47] Yang X. Meng T. (2020). miR-215-5p decreases migration and invasion of trophoblast cells through regulating CDC6 in preeclampsia. Cell Biochem. Funct. 38 (4), 472–479. 10.1002/cbf.3492 31972053

[B48] Yao J. Zhao L. Zhao Q. Zhao Y. Sun Y. Zhang Y. (2014). NF-kappaB and Nrf2 signaling pathways contribute to wogonin-mediated inhibition of inflammation-associated colorectal carcinogenesis. Cell Death Dis. 5, e1283. 10.1038/cddis.2014.221 24901054 PMC4611709

[B49] Yoshida K. Sugimoto N. Iwahori S. Yugawa T. Narisawa-Saito M. Kiyono T. (2010). CDC6 interaction with ATR regulates activation of a replication checkpoint in higher eukaryotic cells. J. Cell Sci. 123, 225–235. 10.1242/jcs.058693 20048340

[B50] Yu X. Liu Y. Yin L. Peng Y. Peng Y. Gao Y. (2019). Radiation-promoted CDC6 protein stability contributes to radioresistance by regulating senescence and epithelial to mesenchymal transition. Oncogene 38, 549–563. 10.1038/s41388-018-0460-4 30158672 PMC6345673

[B51] Zhao Q. Wang J. Zou M. J. Hu R. Zhao L. Qiang L. (2010). Wogonin potentiates the antitumor effects of low dose 5-fluorouracil against gastric cancer through induction of apoptosis by down-regulation of NF-kappaB and regulation of its metabolism. Toxicol. Lett. 197, 201–210. 10.1016/j.toxlet.2010.05.019 20570612

[B52] Zhao Y. Yao J. Wu X. P. Zhao L. Zhou Y. X. Zhang Y. (2015). Wogonin suppresses human alveolar adenocarcinoma cell A549 migration in inflammatory microenvironment by modulating the IL-6/STAT3 signaling pathway. Mol. Carcinog. 54 Suppl 1, E81–E93. 10.1002/mc.22182 24976450

[B53] Zhou L. Liu X. D. Sun M. Zhang X. German P. Bai S. (2016). Targeting MET and AXL overcomes resistance to sunitinib therapy in renal cell carcinoma. Oncogene 35, 2687–2697. 10.1038/onc.2015.343 26364599 PMC4791213

[B54] Zou Y. Mi J. Cui J. Lu D. Zhang X. Guo C. (2009). Characterization of nuclear localization signal in the N terminus of CUL4B and its essential role in cyclin E degradation and cell cycle progression. J. Biol. Chem. 284, 33320–33332. 10.1074/jbc.M109.050427 19801544 PMC2785175

[B55] Zou Y. Mi J. Wang W. Lu J. Zhao W. Liu Z. (2013). CUL4B promotes replication licensing by up-regulating the CDK2-CDC6 cascade. J. Cell Biol. 200, 743–756. 10.1083/jcb.201206065 23479742 PMC3601365

