# Supplementary figures and images for: Wogonin Induces Apoptosis and Reverses Sunitinib Resistance of Renal Cell Carcinoma Cells via Inhibiting CDK4-RB Pathway
Source: Front Pharmacol. 2020 Jul 24;11:1152. doi: 10.3389/fphar.2020.01152 (PMC7394056; doi:10.3389/fphar.2020.01152)

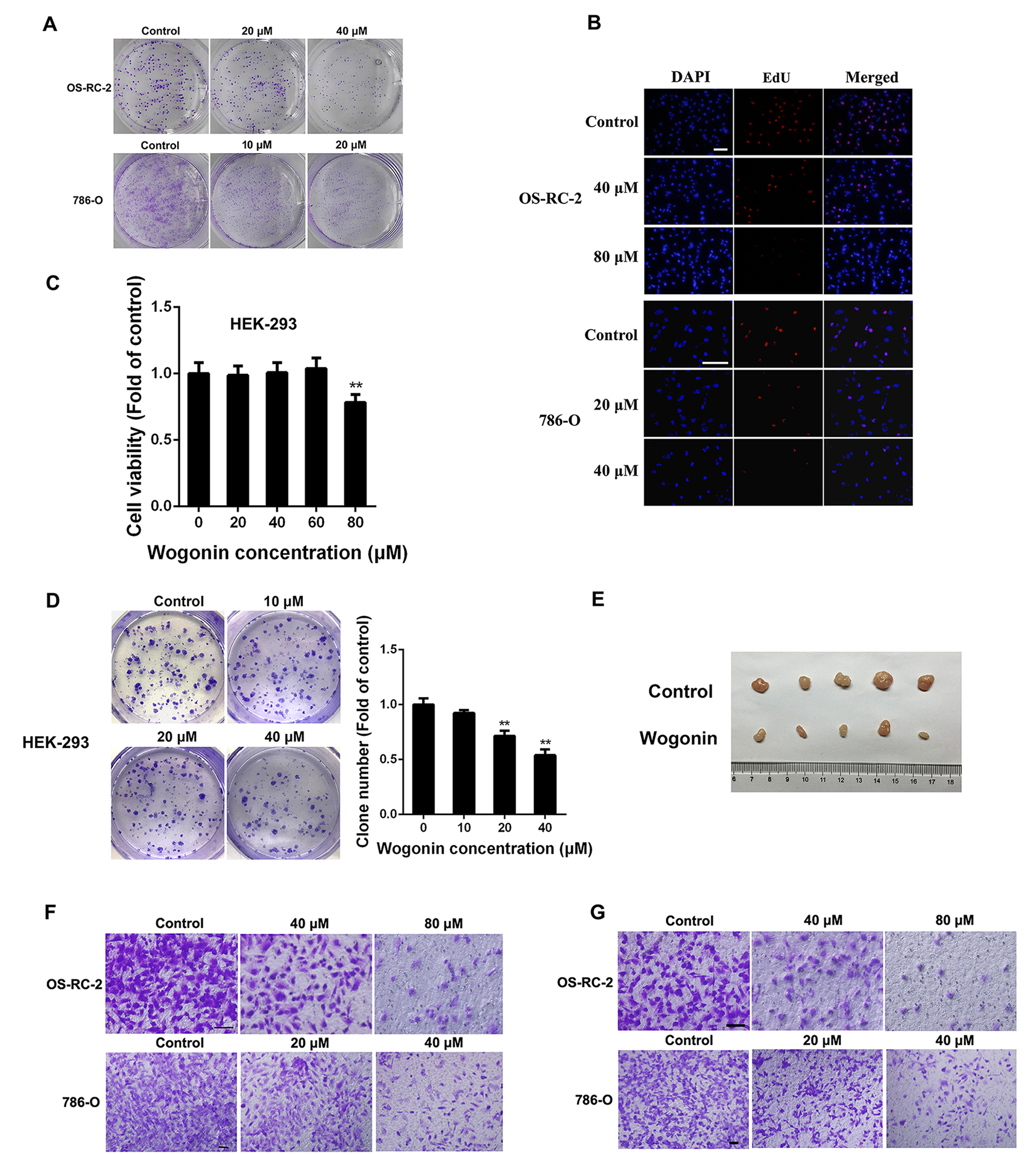

Supplement: Figure S1 — Wogonin inhibits proliferation, migration and invasion of RCC cells. A. Colony formation efficiency of OS-RC-2 and 786-O cells treated with or without wogonin. B. EdU incorporation assays of OS-RC-2 and 786-O cells treated with or without different concentrations of wogonin for 24 h (scale bar, 100 μm). C. MTT assays of HEK-293 cells treated with different concentrations of wogonin for 48 h. D. Colony formation efficiency of HEK-293 cells treated with or without wogonin. E. Tumor formation by 786-O cells in nude mice. 1×10^6^ 786-O cells were injected subcutaneously into nude mice. Mice were either treated with wogonin (40 mg/kg) or treated with vehicle (as control) everyday for 2 weeks. F–G. Cells were treated with different concentrations of wogonin for 24 h, and transwell assays were performed to evaluate the ability of cell migration (F) and invasion (G) (scale bar, 50 μm). [file Image_1.tif]

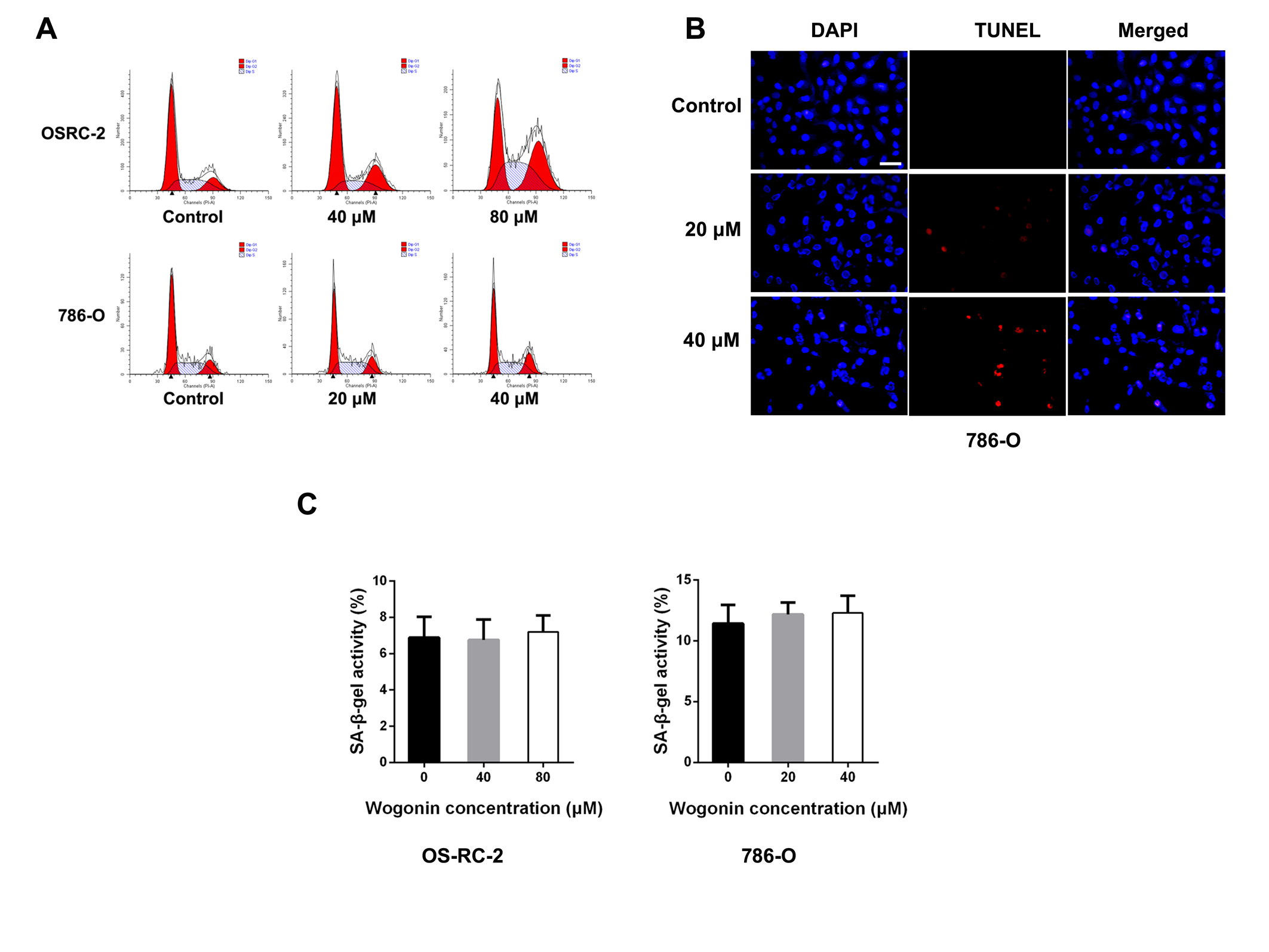

Supplement: Figure S2 — Wogonin induces apoptosis of RCC cells. A. OS-RC-2 and 786-O cells were treated with or without different concentrations of wogonin for 24 h and cell cycle distribution was determined by flow cytometry. B. TUNEL assays of 786-O cells treated with or without wogonin at different concentrations for 24h (scale bar, 50 μm). C. OS-RC-2 and 786-O cells were treated with or without different concentrations of wogonin for 48 h and SA-β-gal activity assays were performed. [file Image_2.tif]

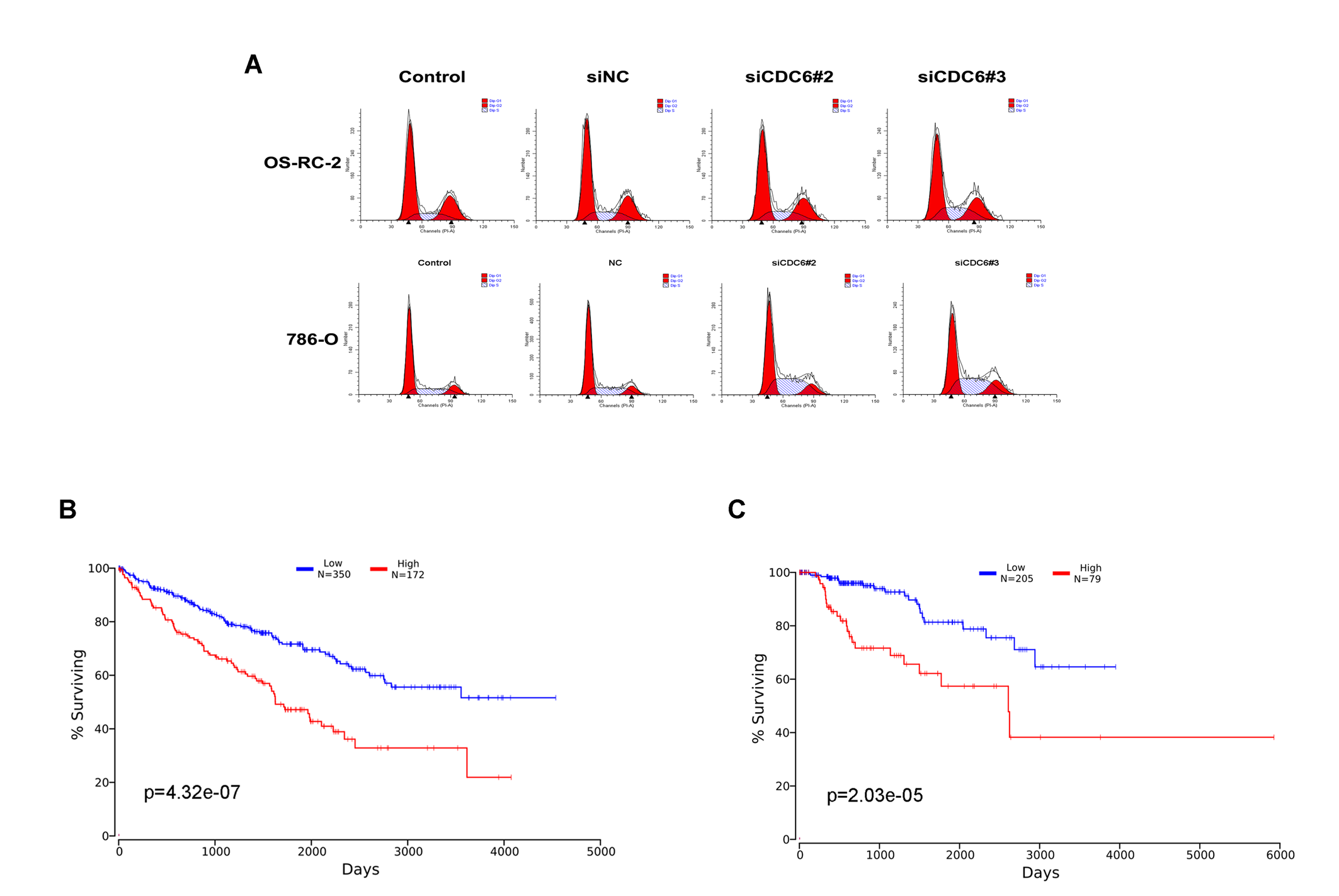

Supplement: Figure S3 — CDC6 expression is correlated with cell cycle of RCC cells and prognosis of RCC patients. A. Cells were transfected with indicated siRNAs for 72 h. Cell cycle distribution was determined by flow cytometry. B. Kaplan–Meier survival analysis for CDC6 expression in renal clear cell carcinoma patients from Oncolnc. C. Kaplan–Meier survival analysis for CDC6 expression in renal papillary cell carcinoma patients from Oncolnc. [file Image_3.tif]

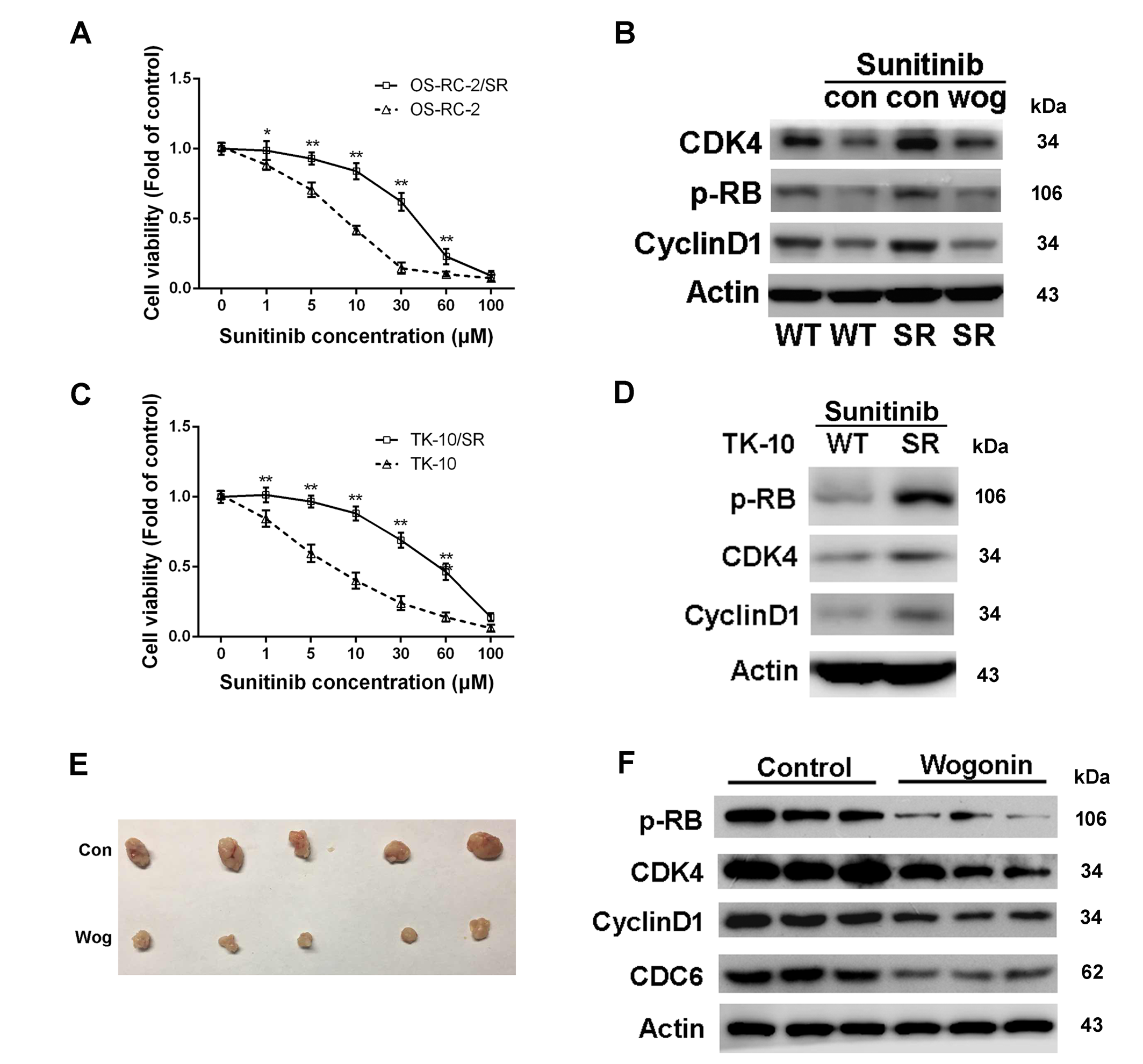

Supplement: Figure S4 — Wogonin suppresses CDK4-RB pathway in sunitinib resistant RCC cells and inhibits proliferation of 786-O/SR cells in vivo. A. Wildtype (WT) or sunitinib-resistant (SR) OS-RC-2 cells were treated with different concentrations of sunitinib for 48 h and cell viability was measured by MTT assays. B. OS-RC-2 or OS-RC/SR cells were treated with 10 μM sunitinib alone or together 40 μM wogonin for 24 h. Indicated protein levels were determined by Western blot. C. Wildtype (WT) or sunitinib-resistant (SR) TK-10 cells were treated with different concentrations of sunitinib for 48 h and cell viability was measured by MTT assays. D. The protein levels of CDK4, p-RB and Cylcin D1 were determined by Western blot in TK-10/WT and TK-10/SR cells after being treated with 10 μM sunitinib for 24 h. E. Tumor formation by 786-O/SR cells in nude mice. 1×10^6^ 786-O/SR cells were injected subcutaneously into nude mice. Mice were either treated with wogonin (40 mg/kg) and sunitinib (20 mg/kg) or treated with sunitinib (20 mg/kg) alone (as control) everyday for 2 weeks. F. The expression of CDK4, p-RB, CyclinD1 and CDC6 in tumor tissues were determined by Western blot. [file Image_4.tif]
