# Supplementary material for: Wogonin Induces Apoptosis and Reverses Sunitinib Resistance of Renal Cell Carcinoma Cells via Inhibiting CDK4-RB Pathway
Source: Front Pharmacol. 2020 Jul 24;11:1152. doi: 10.3389/fphar.2020.01152 (PMC7394056; doi:10.3389/fphar.2020.01152)
Supplement: Supplementary file 5 [file Table_1.docx]

**Supplementary Table 1. Sequence of siRNAs**

| **Name** | **Sequence （5’ - 3’）** |
| --- | --- |
| siNC  siCDC6#1  siCDC6#2  siCDC6#3  siRB#1  siRB#2 | UUCUUCGAACGUGUCACGUTT  GACAATCAGCTGACAATTA  AGGCACTTGCTACCAGCAA  CCAAGAAGGAGCACAAGAT  GGAAAGGACAUGUGAACUUTT  GCAGAAGGCAACUUGACAATT |
