# Supplementary material for: Wogonin Induces Apoptosis and Reverses Sunitinib Resistance of Renal Cell Carcinoma Cells via Inhibiting CDK4-RB Pathway
Source: Front Pharmacol. 2020 Jul 24;11:1152. doi: 10.3389/fphar.2020.01152 (PMC7394056; doi:10.3389/fphar.2020.01152)
Supplement: Supplementary file 6 [file Table_2.docx]

**Supplementary Table 2. Antibodies used in immunofluorescence staining and Western Blot**

| **Name** | **Corportation** | **Cat Number** |
| --- | --- | --- |
| rabbit anti-γH2A.X  rabbit anti-CDH1  rabbit anti-CDH2  rabbit anti-Vimentin  rabbit anti-cleaved caspase-3  rabbit anti-cleaved PARP  rabbit anti-CDC6  rabbit anti-MCM2  rabbit anti-MCM3  rabbit anti-MCM4  mouse anti-β-actin  rabbit anti-MCM6  mouse anti-ORC2  rabbit anti-PCNA  rabbit anti-H3  rabbit anti-CDK4  rabbit anti-CDK6  rabbit anti-p-RB  rabbit anti-RB  rabbit anti-Cyclin D1 | Cell Signaling Technology  Cell Signaling Technology  Cell Signaling Technology  Cell Signaling Technology  Cell Signaling Technology  Cell Signaling Technology  Cell Signaling Technology  Cell Signaling Technology  Cell Signaling Technology  Cell Signaling Technology  Santa Cruz Biotechnology  Abcam  Abcam  Abcam  Abcam  Abcam  Abcam  Abcam  Abcam  Abcam | [9718](https://www.cst-c.com.cn/products/primary-antibodies/phospho-histone-h2a-x-ser139-20e3-rabbit-mab/9718?N=4294956287&Ntt=h2ax&fromPage=plp)  3195  13116  5741  9661  5625  3387  3619  4012  12973  sc-58673  ab4458  ab31930  ab18197  ab1791  ab108357  ab124821  ab47763  ab181616  ab134175 |
