# Supplementary material for: Wogonin Induces Apoptosis and Reverses Sunitinib Resistance of Renal Cell Carcinoma Cells via Inhibiting CDK4-RB Pathway
Source: Front Pharmacol. 2020 Jul 24;11:1152. doi: 10.3389/fphar.2020.01152 (PMC7394056; doi:10.3389/fphar.2020.01152)
Supplement: Supplementary file 7 [file Table_3.docx]

**Supplementary Table 3. Primers used for real-time PCR**

| **Name** | **Forward primer (5'→3')** | **Reverse primer (5'→3')** |
| --- | --- | --- |
| **CDC6**  **CDK4**  **CyclinD1**  **CDC25A**  **CCNA2**  **CCNE2** | GCCGAACTAGAACAGCATCTT  GGGGACCTAGAGCAACTTACT  CTCTGTGGAAGGCAGTTCAAA  GTGAAGGCGCTATTTGGCG  CGCTGGCGGTACTGAAGTC  TCAAGACGAAGTAGCCGTTTAC | GCCGAACTAGAACAGCATCTT  CAGCGCAGTCCTTCCAAAT  TCAGGTAATCCCACCTTGTGTT  TGGTTGCTCATAATCACTGCC  GAGGAACGGTGACATGCTCAT  TGACATCCTGGGTAGTTTTCCTC |
